# Evaluation of dried blood spot sampling for verification of exposure to chemical threat agents

**DOI:** 10.1007/s11419-025-00721-8

**Published:** 2025-04-15

**Authors:** Katie A. Walker, Trinity K. Rudd, Justin N. Vignola, Thomas M. McClymont, Noah D. Roberts, Kevin Laitipaya, Robert C. diTargiani

**Affiliations:** United States Army Medical Research Institute of Chemical Defense, 8350 Ricketts Point Road, Aberdeen Proving Ground, MD, 21010 USA

**Keywords:** Liquid chromatography, Mass spectrometry, Dried blood spot, Nerve agent, Sulfur mustard, Opioid

## Abstract

**Purpose:**

Exposure to chemical threat agents (CTAs), including nerve agents, the vesicating agent sulfur mustard, and opioids, remains a significant threat to warfighter and civilian populations. Definitive analytical methods to verify exposure to CTAs require shipping refrigerated or frozen biomedical samples to reference laboratories for analysis. Logistical and financial burdens arise as the transport of biomedical samples is subject to strict restrictions and complex packaging, which, if done incorrectly, can lead to sample deterioration. The use of dried blood spot (DBS) sampling could provide operational improvements for collecting, storing, and shipping important forensic samples. Therefore, this effort focuses on developing DBS techniques with Mitra® 30-µL volumetric absorptive microsampling (VAMS®) devices for use in CTA exposure verification.

**Methods:**

VAMS® devices were loaded and dried with human whole blood that was exposed to the metabolites pinacolyl methylphosphonic acid (PMPA), ethyl methylphosphonic acid (EMPA), 1,1’sulfonylbis[2-(methylsulfinyl)ethane] (SBMSE), norfentanyl, norcarfentanil, norsufentanil, and norlofentanil. Following extraction from the VAMS® devices, metabolites were detected using liquid chromatography-tandem mass spectrometry (LC–MS/MS). The methods were validated for performance by assessing sensitivity, precision, accuracy, and recovery.

**Results:**

These methods were sensitive to 1 ng/mL for SBMSE, 0.5 ng/mL for PMPA, EMPA, and norfentanyl; 0.1 ng/mL for norlofentanil, and 0.05 ng/mL for norsufentanil and norcarfentanil. All methods met acceptable precision and accuracy criteria with favorable recovery.

**Conclusions:**

These results demonstrated the utility of VAMS® in stabilizing human whole blood and show promise as an improved collection method for verification of exposure to various CTAs.

**Supplementary Information:**

The online version contains supplementary material available at 10.1007/s11419-025-00721-8.

## Introduction

Organophosphorus nerve agents (OPNAs) are potent and irreversible inhibitors of acetylcholinesterase (AChE). Inhibition of AChE activity results in an accumulation of the neurotransmitter acetylcholine, and thereby overstimulation of the cholinergic system [1, 2]. Signs and symptoms of exposure include excessive salivation, lacrimation, miosis, hypotension, muscle fasciculations, severe seizures that can lead to *status epilepticus*, and fatal respiratory complications [2–4]. Although first developed in the 1930s, OPNAs are still employed in warfare and terrorism and remain a significant threat to the warfighter and civilian populations [2, 3, 5, 6].

Several biomarkers have been studied for the verification of OPNA exposure, including the alkyl methylphosphonic acid (AMPA) metabolites. The AMPA metabolites readily form by hydrolysis in the body, and each OPNA has an initial unique hydrolysis product [7]. Specifically, pinacolyl methylphosphonic acid (PMPA) and ethyl methylphosphonic acid (EMPA) are the intermediate hydrolysis degradation products of the OPNAs GD and VX, respectively [6, 8].

The AMPA metabolites are attractive biomarkers for exposure verification as they require fewer reagent expenses and less laborious sample preparation procedures compared to other OPNA plasma biomarkers, such as those involving adducted peptides. This is especially true when detection is carried out using liquid chromatography-tandem mass spectrometry (LC–MS/MS), as opposed to gas chromatography techniques, which require chemical derivatization and additional sample preparation procedures [8, 9]. In addition, although the OPNA hydrolysis products are excreted via urine, they are widely disseminated in the body in blood and tissue and were detected in blood following both human and animal exposures to VX, GB, and GF [7, 9, 10]. Concentrations ranging from 2 to 135 ng/mL of isopropyl methylphosphonic acid, the hydrolyzed product of GB, were measured in human serum samples collected between 1.5 and 2.5 h post-exposure, and other reports confirmed that the AMPA metabolites remain in the blood for days following exposure [7, 10, 11].

Sulfur mustard (HD; bis-(2-chloroethyl) sulfide) is a toxic blistering agent that poses a serious incapacitating threat to humans [12]. Depending on the extent of exposure, toxic effects can range from skin erythema to skin blisters, ocular injury, respiratory tract damage, and death [12–14]. Initially deployed as a chemical weapon in 1917, HD was used in several military conflicts and remains a major health threat to civilians due to its easy production and existing stockpiles [12–16].

A major metabolic pathway of sulfur mustard includes the action of β-lyase on glutathione-derived conjugates of HD to form 1,1’sulfonylbis[2-(methylsulfinyl)ethane] (SBMSE) and 1-methylsulfinyl-2-[2-(methylthio)-ethylsulfonyl]ethane (MSMTESE) [16, 17]. 1-methylsulfinyl-2-[2-(methylthio)-ethylsulfonyl)ethane] (SBMTE) is a reduced form of both of these metabolites and can be generated during sample preparation to increase signal. These β-lyase metabolites are definitive, unambiguous biomarkers of HD exposure as they are nonexistent in the unexposed population [12, 17]. These are considered attractive biomarkers for early verification of HD exposure as they exist in plasma and urine for hours or even days after exposure and the LC–MS/MS analysis procedures are simpler and faster compared to those involving HD adducts [12, 14]. Although limited information exists for the β-lyase metabolites as human plasma biomarkers of HD exposure, Li et al*.* demonstrated that SBMSE and MSMTESE were detected and quantitatively analyzed from rat plasma over a 48-h monitoring period following HD exposure within the calibration curve 0.01–500 ng/mL [14]. In addition, Xu et al*.* detected low levels of SBMSE and MSMTESE in blood samples from four human patients within the first week following an accidental HD exposure [16].

Fentanyl is a synthetic primary μ-opioid antagonist and a potent narcotic analgesic [18]. Initially synthesized by Janssen Pharmaceuticals in the 1960s, fentanyl was the first in a family of potent synthetic opioid analgesics developed [19]. Fentanyl is approximately 100-fold more potent than morphine, and modification of fentanyl’s 4-*N*-anilinopiperidine core yielded many novel analogs that retain or improve upon its original potency and rapid onset of action [18, 20]. Multiple analogs of fentanyl were developed for the medical and veterinary field as they have uses in anesthesiology, palliative care, and chronic pain management [18–20]. These analogs include sufentanil, carfentanil and lofentanil. Due to its high potency, fentanyl and its numerous derivatives are frequently misused, resulting in adverse effects such as respiratory depression, muscular rigidity, apnea, overdose, and death, and subsequently are an increasing public health concern [19, 21]. Investigations of fentanyl and its derivatives to the opioid overdose epidemic are currently underway in the United States, and both civilian and military emergency personnel are increasingly concerned with the possibility of unintentional exposures that might occur in the course of their operational duties [20, 22].

In humans, fentanyl is mainly metabolized in the liver into norfentanyl through oxidative *N*-dealkylation by hepatic cytochrome P450 3A4 (CYP3A4) [18, 21, 23, 24]. Like fentanyl, analogs such as sufentanil, carfentanil and lofentanil are primarily metabolized via the CYP3A4 hepatic pathway, generating the *N*-dealkylated products norsufentanil, norcarfentanil and norlofentanil, respectively [21, 23, 24]. An important point to note is that exclusively monitoring metabolites of opioid exposure does not conclusively determine the causative parent drug, as many fentanyl analogs share the same metabolite [20, 23]. However, detection of these major nor-metabolites would be beneficial for verification purposes and additional analyses can be conducted when necessary for causative parent drug identification.

Definitive analytical methods used to verify exposure to chemical threat agents (CTAs) through the detection of the above biomarkers requires shipping refrigerated or frozen biomedical samples, such as urine, blood, or plasma, to a reference laboratory for analysis. This is a logistical burden, especially if samples originate from remote, austere locations. Transporting patient samples is subject to strict restrictions, safety prerequisites, and requires complex packaging, which is important for sample integrity [15]. Furthermore, additional shipping constraints must be fulfilled if dry ice is used for frozen samples [15]. Reference laboratories then have the financial burden of cold storage for these samples and establishing freezer farms for long-term storage. Alternatively, the use of dried blood spot (DBS) sampling is an appealing alternative for bioanalysis because it offers many advantages, including point-of-care, minimally invasive and low-volume collection, decreased infection hazard for clinical personnel, and more economical and convenient shipping and storage that is better suited to the infrastructural constraints of crisis regions and situations [15, 19, 25].

Since 2014, novel DBS technologies have been introduced onto the market, providing new beneficial qualities that improve upon the simple filter paper that was utilized for DBS sampling since the early 1960s [15]. The Mitra® volumetric absorptive microsampling (VAMS®) devices (Neoteryx, Torrance, CA) are an attractive new technology for DBS sampling as they provide all the advantages of DBS shipping while enabling easy sampling with volumetric collection of fixed-sample volumes and a relatively high volume of sample collection among DBS technologies. In addition, VAMS® devices provide the potential for high-throughput analysis if used in a 96-autotrack, which would be favorable in mass casualty situations. Furthermore, the Mitra® device was better suited for quantification because it is not subject to the hematocrit bias found with punched filter paper [15].

This effort focuses on the use of VAMS® technology to stabilize whole blood samples that were exposed to CTA metabolites to investigate the utility of VAMS® devices for exposure verification. VAMS® devices were loaded and dried with human whole blood samples that were exposed to the metabolites PMPA, EMPA, SBMSE, norfentanyl, norcarfentanil, norsufentanil, and norlofentanil. Following extraction off the VAMS® devices, metabolites were detected using LC–MS/MS. These methods were validated for performance, and the results support VAMS® as an improved collection method for verification of exposure to various CTAs.

## Materials & methods

### Chemicals/blood

Human whole blood (pooled gender; lithium heparin) was obtained from BioIVT (Westbury, NY). Optima LC/MS acetonitrile, methanol, water, and formic acid were obtained from Thermo Fisher Scientific (Waltham, MA). Ammonium formate (≥ 99.0%) was obtained from Honeywell Fluka (Charlotte, NC). Ammonium acetate (≥ 99.0%) was obtained from Millipore Sigma (Burlington, MA). Pinacolyl methylphosphonic acid (PMPA), PMPA-*d*3 (deuterated PMPA), ethyl methylphosphonic acid (EMPA), EMPA-*d*3 (deuterated EMPA), norfentanyl oxalate, norcarfentanil oxalate, norcarfentanil-^13^C_6_ oxalate were obtained from Cerilliant (Round Rock, TX). 1,1’sulfonylbis[2-(methylsulfinyl)ethane] (SBMSE) was synthesized in-house at the U.S. Army Medical Research Institute of Chemical Defense (USAMRICD; Aberdeen Proving Ground, MD). SBMSE-*d*6 (deuterated SBMSE) was provided by Defense Science and Technology Laboratory (DSTL; Porton Down, UK). Norsufentanil, norsufentanil-*d*3, and norfentanyl-*d5* were obtained from Cayman Chemical Company (Ann Arbor, MI). Norlofentanil hydrochloride was obtained from Toronto Research Chemicals (Ontario, CA). Mitra® VAMS® devices were purchased from Neoteryx (Torrance, CA).

LC–MS/MS analysis was performed on an Agilent 1290 Infinity LC system (Agilent Technologies, Santa Clara, CA) interfaced to a Sciex 6500 + QTrap triple quadrupole MS system (Sciex, Framingham, MA).

### PMPA & EMPA

#### Calibration curve and quality control standard preparation for method validation

Assay validation was completed using pooled-gender, human whole blood (lithium heparin) to prepare calibration curves, quality control (QC) standards, and blanks. Blood was spiked with PMPA or EMPA and diluted to produce eight calibrators at 0.5, 1, 5, 20, 40, 60, 80, and 100 ng/mL and three QC standards at 2.5, 25, and 75 ng/mL.

Calibrators, QC standards, and blank control samples were all spotted onto VAMS^®^ by first pipetting 35 µL of sample onto parafilm. The sample was then collected off the parafilm using the 30-µL VAMS^®^, as recommended by the manufacturer. All VAMS^®^ were allowed to dry overnight at 25 °C.

After drying, sampler tips were placed into 15-mL tubes before the addition of 1 mL of 1:1 methanol:acetonitrile. PMPA-*d*3 and EMPA-*d*3 served as the internal standard (IS) for PMPA and EMPA, respectively. 10 μL of IS was added to all calibrators and QC standards for a final IS concentration of 1 ng/mL. Then, samples were sonicated for 10 min. After sonication, sampler tips were removed from the extraction solution and the extraction solutions were evaporated under a dry nitrogen stream at 60 °C. Dried samples were reconstituted with 60 µL of 5% water in acetonitrile and analyzed by LC-MS/MS in quadruplicate.

#### Assay instrumentation

LC-MS/MS analysis was performed in negative electrospray ionization mode. Separation was performed on a Halo HILIC column (2.7 µm, 2.1 x 50 mm; Advanced Materials Technology, Wilmington, DE) with a Halo HILIC guard column (2.7 µm, 2.1 x 5 mm; Advanced Materials Technology, Wilmington, DE) at 25 °C using 20 mM ammonium acetate in water (Mobile Phase A) and acetonitrile (Mobile Phase B). The flow rate was 650 µL/min and the injection volume was 5 µL. The elution program was 0–4.0 minutes (10% A), 4.0–4.1 minutes (10–100% A), 4.1–4.5 minutes (100% A), 4.5–4.6 minutes (100–10% A), 4.6–6.5 minutes (10% A). The MS ion source temperature was 650 °C, capillary voltage was − 4.5 kV, curtain gas setting was 30 psi, and the collision assisted dissociation gas was nitrogen (high). Ion source gasses 1 and 2 were set at 50 and 70 psi, respectively. Compound specific parameters for each transition are given in Table S1 in Supplemental Information. The dwell time for all transitions was 75 msec.

### SBMSE

#### Calibration curve and quality control standard preparation for method validation

The method for SBMSE was similar to the AMPA metabolites except the following changes. Blood was spiked with SBMSE and diluted to produce seven calibrators at 1, 5, 20, 40, 60, 80, and 100 ng/mL and three QC standards at 25, 50, and 75 ng/mL.

After drying, sampler tips were placed into 15-mL tubes before the addition of 1 mL of methanol. SBMSE-*d*6 served as the IS and 10 µL of IS was added to all calibrators and QC standards for a final concentration of 1 ng/mL. Dried samples were reconstituted with 60 μL of 0.2-mM ammonium acetate in water and analyzed by LC-MS/MS in quadruplicate.

#### Assay instrumentation

LC-MS/MS analysis was performed in positive electrospray ionization mode. Separation was performed on a Waters Atlantis T3 column (3 µm, 2.1 x 50 mm; Waters Corporation, Milford, MA) at 60 °C using 0.2 mM ammonium formate in water (Mobile Phase A) and 20 mM ammonium formate in methanol (Mobile Phase B). The flow rate was 600 µL/min and the injection volume was 10 µL. The elution program was 0.00–2.50 minutes (100% A), 2.50–2.51 minutes (100–40% A), 2.51–5.50 minutes (40-5% A), 5.50–6.50 minutes (5% A), 6.50–6.51 minutes (5–100% A), 6.51–9.00 minutes (100% A). The MS ion source temperature was 400 °C, capillary voltage was 5.5 kV, curtain gas setting was 20 psi, and the collision assisted dissociation gas was nitrogen (medium). Ion source gasses 1 and 2 were set at 75 and 80 psi, respectively. Compound specific parameters for each transition are given in Table S2 in Supplemental Information. The dwell time for all transitions was 300 msec.

### Norfentanyl, norsufentanil, norcarfentanil and norlofentanil

#### Calibration curve and quality control standard preparation for method validation

The method for the opioid metabolites was similar to the AMPA metabolites except the following changes. Blood was spiked with norfentanyl and diluted to produce eight calibrators at 0.5, 1, 5, 20, 40, 60, 80, and 100 ng/mL and three QC standards at 2.5, 30, and 75 ng/mL. Blood was spiked with norcarfentanil or norsufentanil and diluted to produce ten calibrators at 0.05, 0.1, 0.5, 1, 5, 20, 40, 60, 80, and 100 ng/mL and three QC standards at 2.5, 30, and 75 ng/mL. Blood was spiked with norlofentanil and diluted to produce nine calibrators at 0.1, 0.5, 1, 5, 20, 40, 60, 80, and 100 ng/mL and three QC standards at 2.5, 30, and 75 ng/mL.

After drying, sampler tips were placed into 15-mL tubes before the addition of 1 mL of 1:1 methanol:acetonitrile. Norfentanyl-*d*5 and norcarfentanil-^13^C_6_ served as the IS for norfentanyl and norcarfentanil, respectively. Norsufentanil-*d*3 served as the IS for both norsufentanil and norlofentanil. Ten μL of IS was added to all calibrators and QC standards for a final IS concentration of 5 ng/mL. Dried samples were reconstituted with 60 μL of 0.1% formic acid in water and analyzed by LC-MS/MS in quadruplicate.

#### Assay instrumentation

LC-MS/MS analysis was performed in positive electrospray ionization mode. Separation was performed on an Agilent Pursuit PFP column (3 μm, 2.0 mm x 50 mm; Agilent Technologies, Santa Clara, CA) with an Agilent Pursuit PFP MetaGuard guard column (3 µm, 2.0 x 10 mm; Agilent Technologies, Santa Clara, CA) at 25 ℃ using 0.1% formic acid in water (Mobile Phase A) and 0.1% formic acid in acetonitrile (Mobile Phase B). The flow rate was 450 μL/min and the injection volume was 10 μL. The elution program was 0.0–0.5 minutes (90% A), 0.5–0.8 minutes (90–80% A), 0.8–1.5 minutes (80% A), 1.5–4.0 minutes (80–50% A), 4.0-4.1 minutes (50–5% A), 4.1–7.0 minutes (5% A), 7.0–7.5 minutes (5–90% A), 7.5–10.0 minutes (90% A). The MS ion source temperature was 450 °C, capillary voltage was 5.5 kV, curtain gas setting was 30 psi, and the collision assisted dissociation gas was nitrogen (low). Ion source gasses 1 and 2 were both set at 60 psi. Compound specific parameters for each transition are given in Table S3 in Supplemental Information. Dwell time was set at 20 ms for norfentanyl and norcarfentanil mass transitions and 30 ms for norlofentanil and norsufentanil mass transitions.

### Data processing

Data were processed using Analyst software (version 1.7.2) (Sciex, Framingham, MA). Quantification of all data was performed through comparison of the integrated area under the peak of the quantitative ion transition for the target analyte of the multiple reaction monitoring (MRM) chromatogram relative to the integrated area under the peak of the quantitative ion transition for the IS. All chromatographic data were fit to a linear regression, weighted at 1/*y*. GraphPad Prism 9 software was used to prepare all graphical representations.

### Method validation

The methods were validated for performance by assessing linearity, sensitivity, precision, accuracy, and extraction recovery.

The standard calibration curve was generated by plotting the ratio of analyte/IS response versus the theoretical concentrations of the standard calibration curve solutions. A linear regression equation was calculated by method of least-squares. The generated curves were weighted (1/*y*), and the regression coefficients were calculated. Linearity greater than 0.99 was required.

To evaluate interday precision and accuracy, a calibration curve and QC standards were prepared and analyzed on five separate days. Precision was calculated as the standard deviation (SD)/mean interpolated concentration, expressed as percent coefficient of variation (% CV). Accuracy was calculated by (interpolated concentration – theoretical concentration)/theoretical concentration, expressed as a percentage. The criteria for accuracy (% error) was based on an average interpolated concentration ± 15% of the theoretical concentration, except for the lower limit of quantitation (LLOQ) which had a tolerance of ± 20% of the theoretical concentration. For precision, the % CV was required to be ≤ 15%, except for the LLOQ which was required to be ≤ 20%.

Intraday precision and accuracy were evaluated by analyzing a calibration curve and five sets of QC standards in one day. All QC standards were quantified from the regression equation generated from the calibration curve on that individual day. Precision and accuracy values were calculated as described above for the interday study. The acceptable criteria for accuracy were based on an average interpolated QC concentration ± 15% of the theoretical concentration, and acceptable criteria for precision was required to be ≤ 15%.

Recovery was evaluated by comparing the results of extracted samples with extracts of blanks spiked with analyte post-extraction. For each method, a zero-calibrator (blank plus IS) for each QC level was extracted and spiked post-extraction with an amount of analyte corresponding to each QC standard concentration. Extraction recovery was measured by comparing the ratio of analyte/IS response of the zero-calibrator spiked with analyte to the ratio of analyte/IS response of extracted QC standards, expressed as a percentage.

## Results

### PMPA & EMPA

This method detected the presence of PMPA and EMPA as low as 0.5 ng/mL, which is the lowest detection limit compared to other DBS methods for the AMPA hydrolysis products.

No peaks were detected at corresponding retention times for PMPA, EMPA, or their respective IS in 10 unexposed blood samples prepared, extracted, and analyzed.

The methods for PMPA and EMPA were validated for linearity, precision, accuracy, and extraction efficiency. Fig. S1 in Supplemental Information shows a representative calibration curve for PMPA and EMPA. All standard calibration curves were linear from 0.5 to 100 ng/mL. For PMPA, the interday correlation coefficients (*r*^*2*^) ranged from 0.9943 to 0.9998 with a mean *r*^*2*^ value of 0.9982. The intraday *r*^*2*^ value was 0.9998. For EMPA, the interday correlation coefficients ranged from 0.9963 to 0.9993 with a mean *r*^*2*^ value of 0.9983. The intraday *r*^*2*^ value was 0.9990.

A calibration curve was prepared and analyzed on five individual days to determine interday precision and accuracy and one calibration curve with five replicates of the QC standards was prepared and analyzed in one day to determine intraday precision and accuracy. Table S4, in Supplemental Information, shows a summary of precision and accuracy of the interday calibration curve and QC standard replicates for PMPA and EMPA, and Table 1 shows a summary of precision and accuracy of the intraday QC standard replicates for all metabolites. The methods for PMPA and EMPA were accurate with all percent errors within ± 15% of the theoretical concentration, including the LLOQ which had a tolerance of ± 20%. The percent errors fell between − 4.1 and 4.9% for the interday study and − 0.5 to 5.8% for the intraday study. In addition, the methods for PMPA and EMPA were precise with all percent coefficient variations below 15%, including the LLOQ which was required to be below 20%. The coefficients of variation ranged from 0.8 to 6.9% for the interday study and 1.4–4.6% for the intraday study.

**Table 1:** Intraday precision and accuracy (n = 5)

	Theoretical concentration (ng/mL)	Mean interpolated concentration ^a^ (ng/mL)	Accuracy ^b^ (% error)	Precision ^c^ (% CV)
PMPA	2.5	2.64 ± 0.09	5.8	3.6
	25	25.6 ± 0.5	2.6	1.9
	75	78 ± 4	4.5	4.6
EMPA	2.5	2.64 ± 0.04	5.6	1.4
	25	25.5 ± 0.7	2.1	2.7
	75	75 ± 2	– 0.5	2.7
SBMSE	25	24.8 ± 0.6	– 0.7	2.6
	50	51 ± 2	2.2	3.9
	75	78 ± 3	3.9	3.8
Norfentanyl	2.5	2.71 ± 0.07	8.4	2.5
	30	30 ± 1	– 0.1	3.4
	75	77 ± 2	2.6	3.0
Norcarfentanil	2.5	2.49 ± 0.08	– 0.4	3.2
	30	29.0 ± 0.8	– 3.3	2.8
	75	79 ± 4	5.0	5.4
Norsufentanil	2.5	2.77 ± 0.06	10.9	2.2
	30	32 ± 1	7.7	3.2
	75	81 ± 2	8.4	3.0
Norlofentanil	2.5	2.7 ± 0.1	7.2	4.9
	30	32 ± 1	6.0	3.6
	75	82 ± 3	8.9	3.5

^a^Values are expressed as the mean ± SD

^b^Accuracy (% error) = ((interpolated concentration – theoretical concentration) / theoretical concentration) × 100

^c^Precision (% CV) = (SD / mean) × 100

Extraction recovery was evaluated by comparing the area under the curve (AUC) ratio of a zero-calibrator spiked with analyte post-extraction to the AUC ratio of an extracted QC standard. Table 2 shows the percent recovery for the extraction methods for all metabolites. The extraction recoveries ranged from 69–71% for PMPA and 97–105% for EMPA.

**Table 2:** Percent compound recovery (mean ± SD, %). Data at each concentration represent mean AUC ratio of extracted QC standard (n = 4)/mean AUC ratio of the zero-calibrator spiked with analyte post-extraction (n = 4), expressed as a percent

	LQC	MQC	HQC
PMPA	69 ± 4	71 ± 4	69 ± 2
EMPA	105 ± 6	98 ± 2	97.5 ± 0.9
SBMSE	82 ± 4	80 ± 4	81 ± 4
Norfentanyl	64 ± 5	54 ± 1	48 ± 1
Norcarfentanil	47 ± 2	53 ± 3	56 ± 2
Norsufentanil	46 ± 2	36 ± 1	37 ± 1
Norlofentanil	62 ± 1	54 ± 1	52.7 ± 0.8

*LQC* low QC standard; *MQC* middle QC standard; *HQC* high QC standard

### Sulfur mustard

This method detected SBMSE from a 30-µL microsampler and detected the presence of SBMSE down to 1 ng/mL. The method for SBMSE was validated for linearity, precision, accuracy, and extraction efficiency. Fig. S2 in Supplemental Information shows a representative calibration curve for SBMSE. All standard calibration curves were linear from 1 to 100 ng/mL. The interday correlation coefficients ranged from 0.9966 to 0.9989 with a mean *r*^*2*^ value of 0.9979. The intraday *r*^*2*^ value was 0.9966.

No interference by endogenous entities from human whole blood were detected under the current chromatographic and MS conditions for SBMSE and SBMSE-*d*6.

Five calibration curves were prepared and analyzed on individual days to determine interday precision and accuracy and one calibration curve with five replicates of the QC standards was prepared and analyzed in one day to determine intraday precision and accuracy. Table 3 shows a summary of precision and accuracy of the interday calibration curve and QC standard replicates for SBMSE and Table 1 shows a summary of precision and accuracy of the intraday QC standard replicates for all metabolites. The method for SBMSE was accurate with all percent errors within ± 15% of the theoretical concentration. The percent errors fell between − 2.1 to 3.1% for the interday study and − 0.7 to 3.9% for the intraday study. In addition, the method was precise with all percent coefficient of variations below 15%. The % CV ranged from 1.9 to 8.0% for the interday study and 2.6–3.9% for the intraday study.

**Table 3:** SBMSE interday precision and accuracy (n = 5)

	Theoretical concentration (ng/mL)	Mean interpolated concentration ^a^ (ng/mL)	Accuracy ^b^ (% error)	Precision ^c^ (% CV)
SBMSE calibration standards	1	0.98 ± 0.06	– 2.1	6.0
	5	5.0 ± 0.2	– 0.7	3.5
	20	20.6 ± 0.6	3.1	3.0
	40	40 ± 2	0.2	4.5
	60	61 ± 1	1.3	1.9
	80	80 ± 2	0.1	2.6
	100	99 ± 2	– 1.4	2.5
SBMSE QC standards	25	25 ± 2	– 1.6	8.0
	50	50 ± 1	– 0.1	2.5
	75	76 ± 4	0.9	5.7

^a^Values are expressed as the mean ± SD

^b^Accuracy (% error) = ((interpolated concentration – theoretical concentration) / theoretical concentration) × 100

^c^Precision (% CV) = (SD / mean) × 100

Extraction efficiency was evaluated by comparing the AUC ratio of a zero-calibrator spiked with analyte post-extraction to the AUC ratio of an extracted QC standard. Table 2 shows the percent recovery for the extraction methods for all metabolites. The percent recovery was 82, 80, and 81% for the low, middle, and high QC standard, respectively.

### Norfentanyl, norcarfentanil, norsufentanil, and norlofentanil

This method detected the presence of norcarfentanil and norsufentanil as low as 0.05 ng/mL, norlofentanil as low as 0.1 ng/mL, and norfentanyl as low as 0.5 ng/mL. No peaks were detected at corresponding retention times for norfentanyl, norcarfentanil, norsufentanil, norlofentanil, or their respective IS.

The method was validated for linearity. Fig. S3 in Supplemental Information shows a representative calibration curve for norfentanyl, norcarfentanil, norsufentanil, and norlofentanil. For norfentanyl, all standard calibration curves were linear from 0.5 to 100 ng/mL. The interday correlation coefficients ranged from 0.9969 to 0.9988 with a mean *r*^*2*^ value of 0.9981. The intraday *r*^*2*^ value was 0.9988. For norcarfentanil and norsufentanil, all standard calibration curves were linear from 0.05 to 100 ng/mL. The interday correlation coefficients ranged from 0.9976 to 0.9993 for norcarfentanil with a mean *r*^*2*^ value of 0.9987. The intraday *r*^*2*^ value was 0.9989. For norsufentanil, the interday *r*^*2*^ values ranged from 0.9974 to 0.9984 with a mean interday *r*^*2*^ value of 0.9979 and an intraday *r*^*2*^ value of 0.9974. For norlofentanil, all standard calibration curves were linear from 0.1 to 100 ng/mL. The interday correlation coefficients ranged from 0.9945 to 0.9987 with a mean interday *r*^*2*^ value of 0.9974 and an intraday *r*^*2*^ value of 0.9908.

Table 4 shows a summary of precision and accuracy of the interday calibration curve and QC standard replicates for all opioid metabolites and Table 1 shows a summary of precision and accuracy of the intraday QC standard replicates for all analytes.

**Table 4:** Opioid metabolite interday precision and accuracy (n = 5)

	Theoretical concentration (ng/mL)	Mean interpolated concentration ^a^ (ng/mL)	Accuracy ^b^ (% error)	Precision ^c^ (% CV)
Norfentanyl calibration standards	0.5	0.49 ± 0.03	– 2.6	5.8
	1	1.03 ± 0.03	3.2	3.0
	5	5.1 ± 0.1	1.9	2.8
	20	20 ± 1	– 2.3	5.1
	40	39.5 ± 0.8	– 1.2	1.9
	60	58 ± 1	– 3.8	2.1
	80	84 ± 1	5.2	1.3
	100	99 ± 2	– 1.1	1.6
Norfentanyl QC standards	2.5	2.6 ± 0.1	5.9	5.4
	30	29 ± 2	– 3.0	6.0
	75	74 ± 3	– 0.7	3.4
Norcarfentanil calibration standards	0.05	0.053 ± 0.003	6.0	6.3
	0.1	0.101 ± 0.006	1.0	5.9
	0.5	0.51 ± 0.03	1.0	5.3
	1	0.94 ± 0.06	– 5.8	6.4
	5	4.8 ± 0.2	– 3.2	4.7
	20	20.5 ± 0.8	2.3	4.1
	40	40.6 ± 0.9	1.4	2.1
	60	60 ± 2	– 0.5	3.6
	80	80 ± 1	– 0.5	1.7
	100	100 ± 3	– 0.04	2.9
Norcarfentanil QC standards	2.5	2.36 ± 0.09	– 5.5	3.8
	30	29 ± 1	– 4.1	4.2
	75	77 ± 1	2.5	1.8
Norsufentanil calibration standards	0.05	0.051 ± 0.006	1.2	12.1
	0.1	0.105 ± 0.005	5.0	4.7
	0.5	0.473 ± 0.008	– 5.4	1.7
	1	0.98 ± 0.06	– 2.1	6.6
	5	5.3 ± 0.2	5.7	3.7
	20	20 ± 1	– 1.8	5.2
	40	39 ± 2	– 2.5	4.9
	60	60 ± 3	0.3	4.4
	80	81 ± 4	1.3	4.7
	100	100 ± 4	– 0.1	4.5
Norsufentanil QC standards	2.5	2.6 ± 0.2	5.7	6.1
	30	31 ± 1	3.7	4.5
	75	76 ± 2	1.5	2.9
Norlofentanil calibration standards	0.1	0.11 ± 0.01	9.2	9.5
	0.5	0.50 ± 0.03	0.4	6.3
	1	0.96 ± 0.07	– 4.0	6.9
	5	4.8 ± 0.4	– 3.1	7.6
	20	19 ± 1	– 5.1	6.4
	40	41 ± 2	3.1	5.5
	60	58 ± 3	– 3.8	4.3
	80	81 ± 4	1.5	5.4
	100	101 ± 4	0.9	3.5
Norlofentanil QC standards	2.5	2.6 ± 0.2	4.2	6.9
	30	30 ± 2	0.3	6.5
	75	74 ± 4	– 1.4	5.5

^a^Values are expressed as the mean ± SD

^b^Accuracy (% error) = ((interpolated concentration – theoretical concentration) / theoretical concentration) × 100

^c^Precision (% CV) = (SD / mean) × 100

Extraction recovery was evaluated by comparing the AUC ratio of a zero-calibrator spiked with analyte post-extraction to the AUC ratio of an extracted QC standard. Table 2 shows the percent recovery for the extraction methods for all metabolites. The extraction recoveries ranged from 48–64% for norfentanyl, 47–56% for norcarfentanil, 36–46% for norsufentanil, and 52–62% for norlofentanil.

## Discussion

Dried blood samples were evaluated for their utility in verifying human exposure to CTAs. Extraction and LC-MS/MS detection and quantitation protocols for 30-µL dried blood microsamplers were developed and validated for 7 metabolites in human whole blood: PMPA, EMPA, SBMSE, norfentanyl, norcarfentanil, norsufentanil, and norlofentanil.

When developing extraction protocols for all metabolites, the addition of IS was explored. Internal standard is used to improve the precision of quantitative analysis and should be added as early as possible to the sample preparation procedure to account for any variance that occurs during sample preparation. Premixing the blood sample with IS before loading and drying to the microsampler was a viable procedure that resulted in accurate and precise quantitation (data not shown); however, this method is not practical for field application. If the VAMS^®^ devices are intended for use in the field by a warfighter or civilian with no technical training, it is not practical to mix the blood sample with IS at the point of collection. In addition , pre-loading the microsampler with IS would not be practical for field use and would require a small volume of IS as to not saturate the microsampler with the IS solution, which could result in poor accuracy. Furthermore, the addition of IS to a dried microsampler is not possible as the microsampler is already saturated by the blood sample. Alternatively, the addition of IS to the DBS extraction solution during laboratory analysis is more practical for field-use scenarios and resulted in high reproducibility between samples and accurate and precise quantitation. Therefore, the addition of IS to the DBS extraction solution during the laboratory analysis was applied to the extraction protocols for all metabolites.

### PMPA & EMPA

**Fig. 1:**
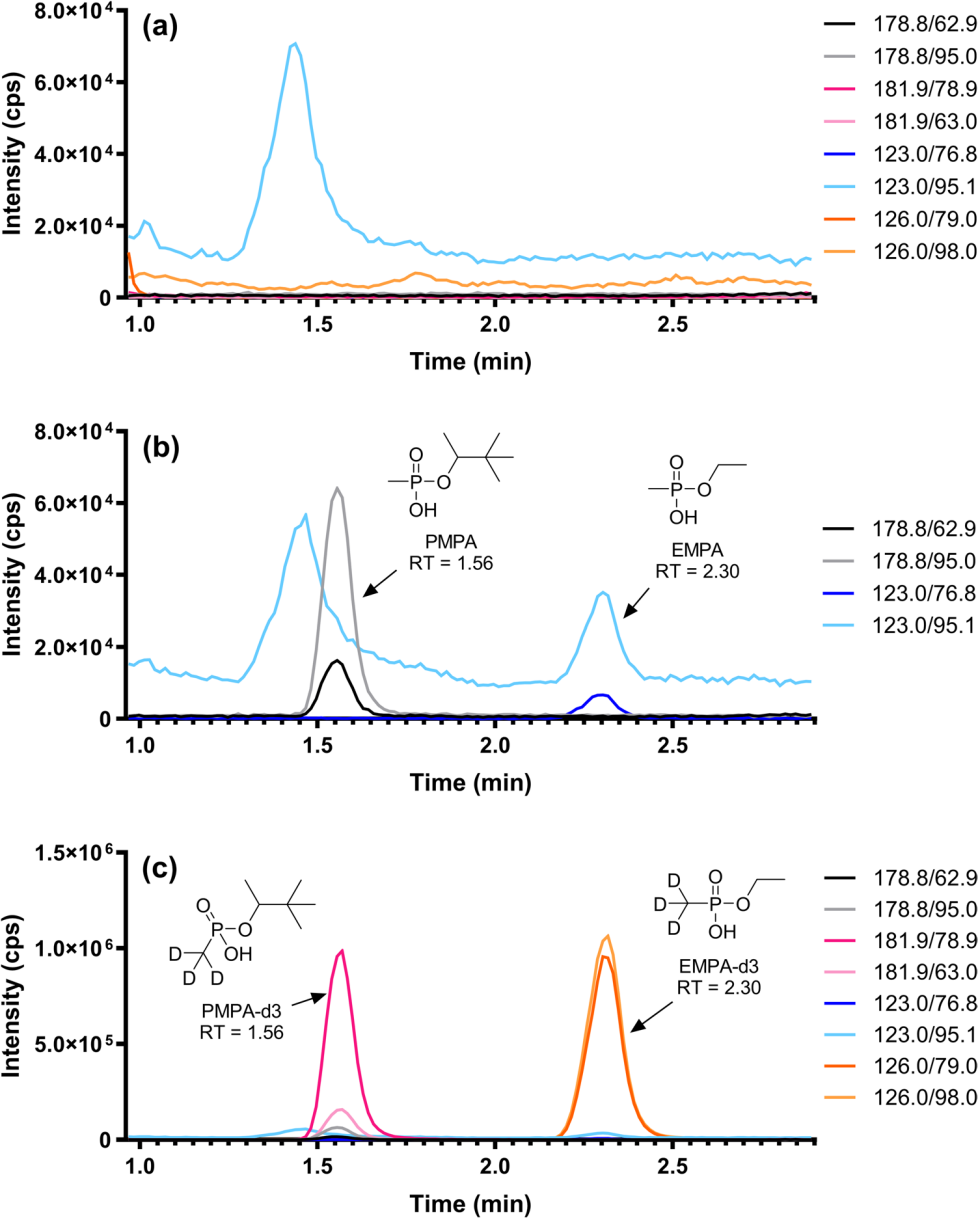
PMPA and EMPA reconstructed ion chromatograms: a representative reconstructed ion chromatogram of a matrix blank (**a**), 0.5 ng/mL PMPA and EMPA (**b**), and 0.5 ng/mL PMPA and EMPA with PMPA-*d*3 and EMPA-*d*3 (c), all following work-up from human whole blood. Both quantitative and qualitative ion transitions are presented for each compound and IS

The methods presented here can detect two metabolites of nerve agent exposure from a 30-µL dried blood microsampler. PMPA and EMPA were chosen not only to be an example of both G-series and V-series agent metabolite detection but also because EMPA historically has the lowest sensitivity among the traditional agent AMPA hydrolysis products. Currently, the only other DBS method for the AMPA hydrolysis products from microsamplers has a 5-ng/mL detection limit from a 10-µL microsampler [7]. Similar to the current method, this method maintained a simple extraction procedure with minimal sample preparation steps allowing for rapid analysis. However, by increasing the sample volume from 10 μL to 30 μL, we increased sensitivity ten-fold from 5 ng/mL to 0.5 ng/mL when extracting from dried blood microsamplers. A representative reconstructed ion chromatogram for the quantitative and qualitative ion transitions of 0.5 ng/mL of PMPA and 0.5 ng/mL of EMPA following work-up from human whole blood is shown in Fig. 1b. Figure 1c depicts the ion chromatogram with their respective IS. In addition, a representative reconstructed ion chromatogram of a matrix blank is shown in Fig. 1a.

Extraction recoveries for PMPA and EMPA were sufficient for quantitation purposes and consistent across all three QC standard concentrations. However, the extraction recovery of PMPA was lower than expected. Since PMPA is more non-polar than EMPA, it was expected that PMPA would have a greater extraction efficiency than EMPA.

### Sulfur mustard

Initial studies investigated SBMSE, MSMTESE, and their common reduced product, SBMTE to determine the best biomarker for verification of exposure to HD using DBS sampling. VAMS® were prepared with MSMTESE alone, SBMSE alone, and MSMTESE and SBMSE together, at 1, 10, and 100 ng/mL.

Native SBMSE and MSMTESE were both detectable at 1 ng/mL, but MSMTESE had a lower signal-to-noise ratio when compared to SBMSE (data not shown). In addition, since native SBMSE was detectable at 1 ng/mL with acceptable signal-to-noise, it was determined that the reduction step was not necessary to increase sensitivity, and its elimination reduced processing time and costs. The sample preparation for the reduced metabolite, SBMTE, required increased reagent costs and was more labor intensive, which decreased throughput and was not compatible for screening large sample quantities. Therefore, SBMSE was selected as the biomarker that would be used to verify exposure to HD using DBS sampling.

**Fig. 2:**
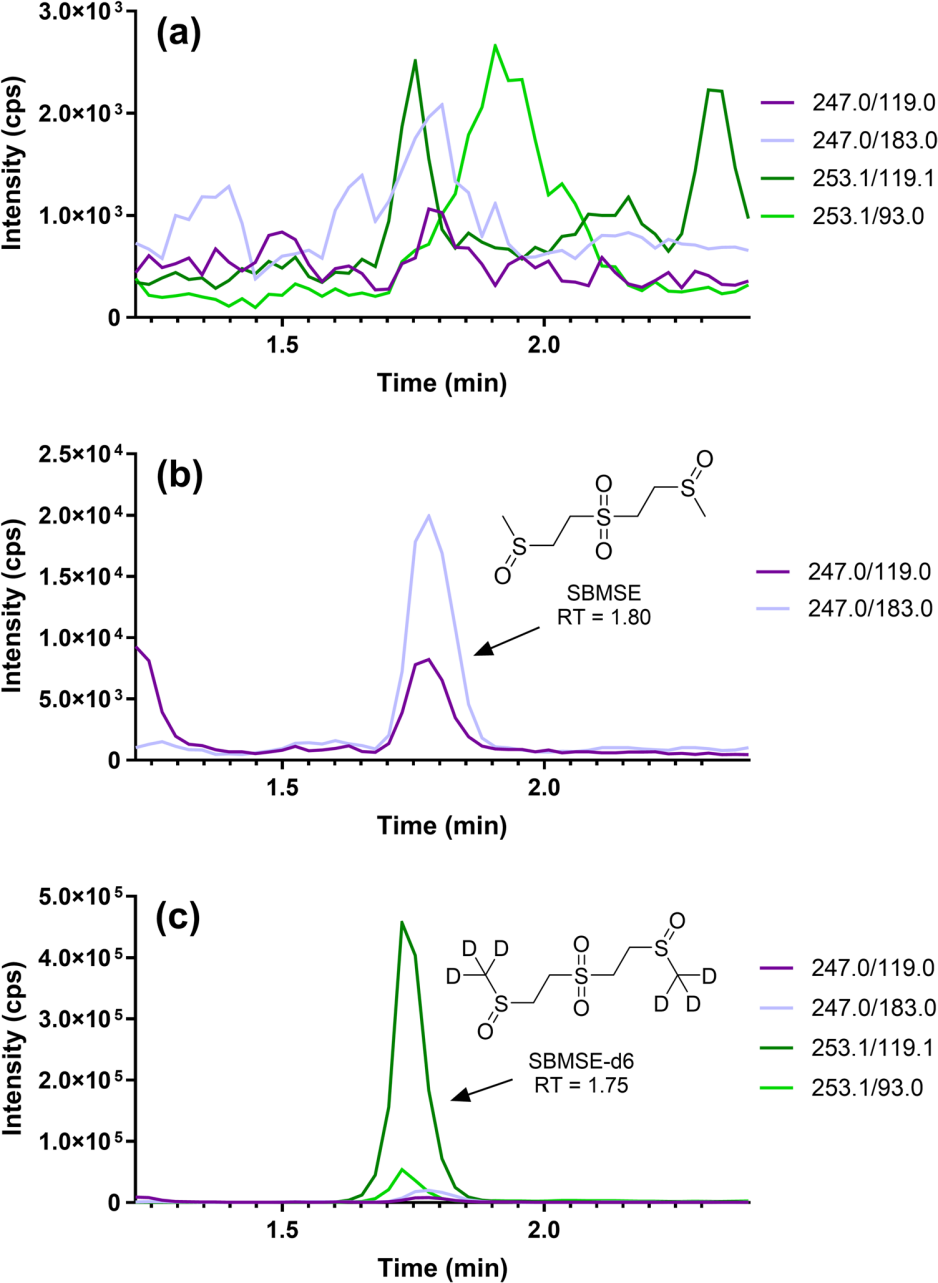
SBMSE reconstructed ion chromatograms: a representative reconstructed ion chromatogram of a matrix blank (**a**), 1 ng/mL SBMSE (**b**), and 1 ng/mL SBMSE with SBMSE-*d*6 (**c**), all following work-up from human whole blood. Both quantitative and qualitative ion transitions are presented for each compound and IS

This method detected SBMSE from a 30 µL microsampler and marks the first time a β-lyase metabolite of HD has been quantified from DBSs. The LLOQ for SBMSE was set at 1 ng/mL, however, it is possible that the LLOQ could be lower, considering 1 ng/mL had good peak area and signal-to-noise. A representative reconstructed ion chromatogram for the quantitative and qualitative ion transitions of 1 ng/mL of SBMSE following work-up from human whole blood is shown in Fig. 2b and Fig. 2c depicts the ion chromatogram of 1 ng/mL SBMSE with IS. In addition, a representative reconstructed ion chromatogram for a matrix blank is presented in Fig. 2a. The extraction recovery of SBMSE was sufficient for quantitation purposes and consistent across all three QC standard concentrations.

### Norfentanyl, norcarfentanil, norsufentanil, and norlofentanil

**Fig. 3:**
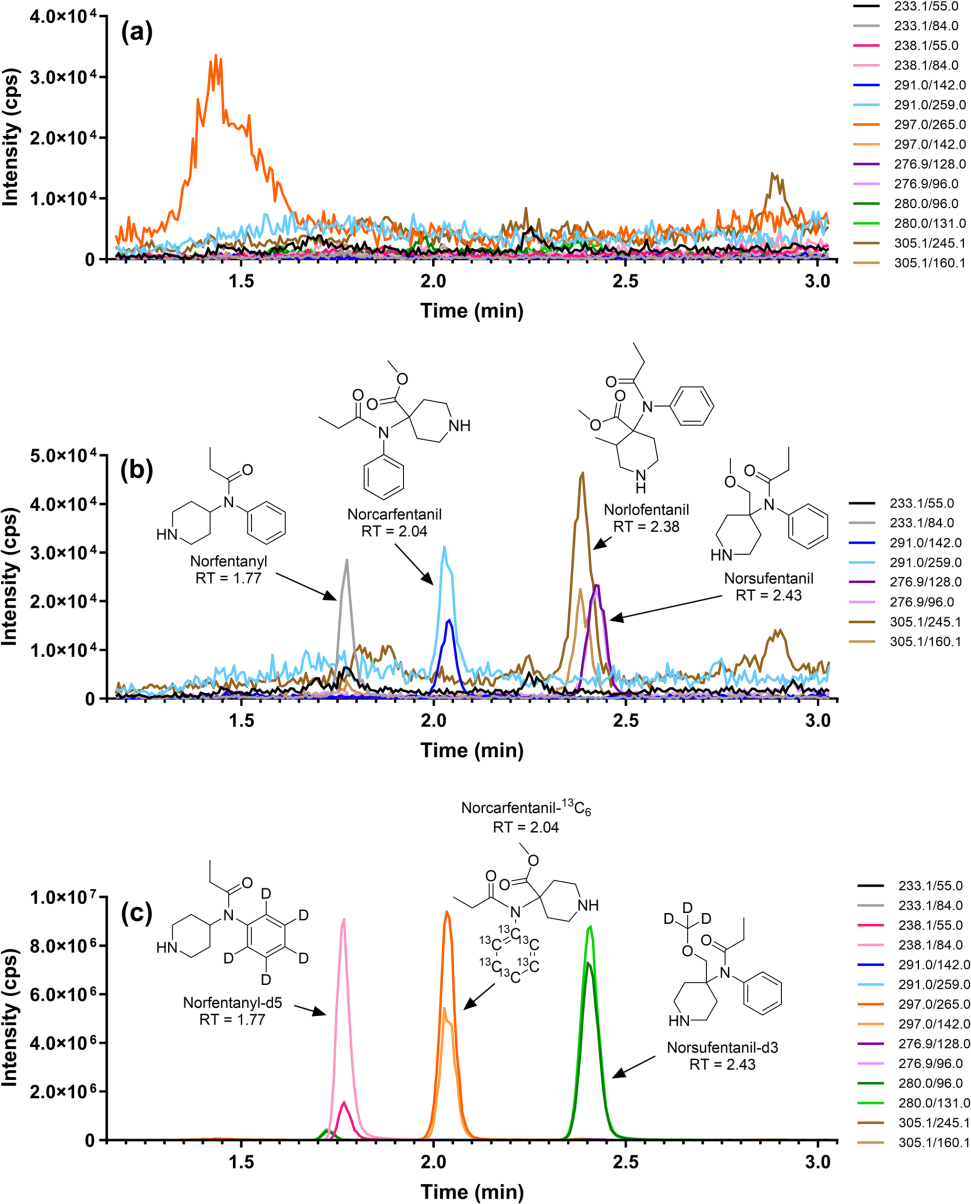
Norfentanyl, norcarfentanil, norsufentanil, and norlofentanil reconstructed ion chromatograms: a representative reconstructed ion chromatogram of a matrix blank (**a**), 0.5 ng/mL norfentanyl, norcarfentanil, norsufentanil, and norlofentanil (**b**), and 0.5 ng/mL norfentanyl, norcarfentanil, norsufentanil, and norlofentanil with norfentanyl-*d*5, norcarfentanil-^13^C_6_, and norsufentanil-*d*3 (**c**), all following work-up from human whole blood. Both quantitative and qualitative ion transitions are presented for each compound and IS

The method presented here detected four biomarkers of opioid exposure from 30-µL dried blood microsamplers. In the case of fentanyl overdose, norfentanyl was detected in blood from 1.4 to 8.9 ng/mL, and in driving under the influence of drugs (DUID) cases in Houston, Texas, between 2019 and 2022, norfentanyl was detected in the blood at a median concentration of 1.7 ng/mL [20, 26]. In addition, Cannaert et al*.* measured 0.532 ng/mL of norcarfentanil in blood following a fatal carfentanil intoxication, Uddayasankar et al*.* measured norcarfentanil concentrations in blood ranging from 0.10 to 3.95 ng/mL between 0–52 h post-emergency room admission in a recreational carfentanil exposure case, and Allibe et al*.* detected norcarfentanil concentrations ranging from 0.05 to 15.8 ng/mL in plasma following carfentanil and remifentanil exposure [27–29]. This suggests that the sensitivities of this method are sufficient for opioid exposure verification purposes. A representative reconstructed ion chromatogram for the quantitative and qualitative ion transitions of 0.5 ng/mL norfentanyl, norcarfentanil, norsufentanil, and norlofentanil following work-up from human whole blood are shown in Fig. 3b. Figure 3c depicts the ion chromatogram with their respective IS. In addition, a representative reconstructed ion chromatogram of a matrix blank is shown in Fig. 3a.

The methods for norfentanyl, norcarfentanil, norsufentanil, and norlofentanil were accurate with all percent errors within ± 15% of the theoretical concentration, including the LLOQ which had a tolerance of ± 20%. The methods for norfentanyl, norcarfentanil, norsufentanil, and norlofentanil were precise with all percent coefficient of variations below 15%, including the LLOQ which was required to be below 20%.

Extraction recoveries for norfentanyl, norcarfentanil, norsufentanil, and norlofentanil were sufficient for quantitation purposes, consistent across all three QC standard concentrations, and consistent between all four opioid metabolites.

These fentanyl analog nor-metabolites have been previously quantified from DBS cards [20]. However, this will be the first validated method extracting norsufentanil, norcarfentanil, and norlofentanil from dried blood microsamplers, and the first time quantifying norfentanyl from 30 µL dried blood microsamplers.

## Conclusion

This study demonstrated the utility of VAMS^®^ in stabilizing human whole blood samples that were exposed to the above metabolites. The LC-MS/MS methods quantified the AMPA metabolites (0.5–100 ng/mL), SBMSE (1–100 ng/mL), and the opioid metabolites (0.5–100 ng/mL), extracted via 30-µL DBSs. The methods for all metabolites were validated for performance, demonstrated acceptable precision and accuracy, with favorable sensitivity and extraction recovery.

VAMS^®^ devices offer a new low-volume, minimally invasive sample collection procedure for field use that is amenable for use by non-technical personnel due to simplified sample collection techniques. Furthermore, the above methods maintained simple extraction procedures with minimal sample preparation steps, allowing for a low-cost, rapid analysis procedure. In the future, this capability can be expanded to include additional CTAs and other common biomedical matrices as well as additional exploration into the stability of target metabolites on VAMS^®^.

## Supplementary Information

Below is the link to the electronic supplementary material.

Supplementary file1 (PDF 267 KB)

## Data Availability

The data presented in this study may be available on request from the corresponding author. The data are not publicly available in a repository at this time but may be requested.
