# Supplementary material for: Evaluation of dried blood spot sampling for verification of exposure to chemical threat agents
Source: Forensic Toxicol. 2025 Apr 15;43(2):280–93. doi: 10.1007/s11419-025-00721-8 (PMC12241275; doi:10.1007/s11419-025-00721-8)
Supplement: Supplementary file 1 — Supplementary file1 (PDF 267 KB) [file 11419_2025_721_MOESM1_ESM.pdf]

Evaluation of dried blood spot sampling for verification of exposure to chemical threat agents: Supplemental Information

Katie A. Walker [1], Trinity K. Rudd [1], Justin N. Vignola [1], Thomas M. McClymont [1], Noah D. Roberts [1], Kevin Laitipaya [1], and Robert C. diTargiani [1,*]

[1] *United States Army Medical Research Institute of Chemical Defense, 8350 Ricketts Point Road, Aberdeen Proving Ground, MD, 21010, USA*

* Correspondence: robert.c.ditargiani.civ@health.mil

Table S1 PMPA, EMPA and IS specific instrument parameters.

	Precursor (*m/z*)	Product (*m/z*)	Declustering Potential (V)	Entrance Potential (V)	Collision Energy (eV)	Collision Exit Potential (V)
PMPA (Quant.)	178.8	62.9	-70.0	-10.0	-85.0	-26.0
PMPA (Qual.)	178.8	95.0	-30.0	-10.0	-26.0	-8.0
PMPA-d3 (Quant.)	181.9	78.9	-85.0	-10.0	-50.0	-10.0
PMPA-d3 (Qual.)	181.9	63.0	-40.0	-10.0	-87.0	-36.0
EMPA (Quant.)	123.0	76.8	-30.0	-10.0	-30.0	-9.0
EMPA (Qual.)	123.0	95.1	-30.0	-15.0	-17.0	-8.0
EMPA-d3 (Quant.)	126.0	79.0	-41.0	-10.0	-33.7	-9.0
EMPA-d3 (Qual.)	126.0	98.0	-32.0	-10.0	-18.0	-11.0

Table S2 SBMSE & SBMSE-d6 specific instrument parameters.

	Precursor (*m/z*)	Product (*m/z*)	Declustering Potential (V)	Entrance Potential (V)	Collision Energy (eV)	Collision Exit Potential (V)
SBMSE (Quant.)	247.0	119.0	85.0	10.0	31.0	16.0
SBMSE (Qual.)	247.0	183.0	85.0	10.0	16.0	22.0
SBMSE-d6 (Quant.)	253.1	119.1	85.0	10.0	30.0	6.6
SBMSE-d6 (Qual.)	253.1	93.0	85.0	10.0	58.0	7.3

Table S3 Norfentanyl, Norcarfentanil, Norsufentanil, Norlofentanil, and IS specific instrument parameters.

	**Precursor (*m/z*)**	**Product (*m/z*)**	**Declustering Potential (V)**	**Entrance Potential (V)**	**Collision Energy (eV)**	**Collision Exit Potential (V)**
Norfentanyl (Quant.)	233.1	55.0	50.0	10.0	54.0	8.0
Norfentanyl (Qual.)	233.1	84.0	40.0	10.0	23.0	13.0
Norfentanyl-d5 (Quant.)	238.1	55.0	40.0	10.0	55.0	23.8
Norfentanyl-d5 (Qual.)	238.1	84.0	50.0	10.0	24.0	10.0
Norcarfentanil (Quant.)	291.0	142.0	50.0	10.0	21.0	20.0
Norcarfentanil (Qual.)	291.0	259.0	50.0	10.0	14.0	18.0
Norcarfentanil-$^{13}C_6$ (Quant.)	297.0	265.0	40.0	10.0	14.0	15.0
Norcarfentanil-$^{13}C_6$ (Qual.)	297.0	142.0	40.0	10.0	22.0	18.0
Norsufentanil (Quant.)	276.9	128.0	40.0	10.0	17.7	16.0
Norsufentanil (Qual.)	276.9	96.0	40.0	10.0	27.0	11.0
Norsufentanil-d3 (Quant.)	280.0	96.0	30.0	10.0	28.0	12.0
Norsufentanil-d3 (Qual.)	280.0	131.0	40.0	10.0	18.4	16.0
Norlofentanil (Quant.)	305.1	245.1	30.0	10.0	19.5	20.0
Norlofentanil (Qual.)	305.1	160.1	30.0	10.0	37.0	19.0

Table S4 PMPA & EMPA interday precision and accuracy (n=5)

	Theoretical Concentration (ng/mL)	Mean Interpolated Concentration [a] (ng/mL)	Accuracy [b] (% error)	Precision [c] (% CV)
**PMPA** Calibration Standards	0.5	0.52 ± 0.03	4.1	5.8
	1	0.984 ± 0.008	-1.6	0.8
	5	5.1 ± 0.2	2.0	4.0
	20	19.6 ± 0.9	-2.2	4.7
	40	38 ± 2	-4.1	6.4
	60	59 ± 2	-2.0	2.9
	80	82 ± 2	1.9	1.9
	100	101 ± 3	1.5	3.1
**PMPA** QC standards	2.5	2.5 ± 0.1	1.6	5.8
	25	24 ± 1	-3.1	5.2
	75	79 ± 5	4.9	6.9
**EMPA** Calibration standards	0.5	0.51 ± 0.03	1.6	5.4
	1	0.98 ± 0.05	-2.0	5.0
	5	5.1 ± 0.1	2.7	2.7
	20	19.8 ± 0.4	-0.9	1.9
	40	38.4 ± 0.5	-4.0	1.4
	60	62 ± 2	3.2	3.2
	80	78 ± 1	-2.2	1.3
	100	102 ± 2	1.6	2.1
**EMPA** QC standards	2.5	2.56 ± 0.05	2.2	2.0
	25	25 ± 1	-1.5	4.0
	75	76 ± 2	1.6	2.7

[a] Values are expressed as the mean ± SD
[b] Accuracy (% error) = ((interpolated concentration – theoretical concentration) / theoretical concentration) × 100
[c] Precision (% CV) = (SD / mean) × 100

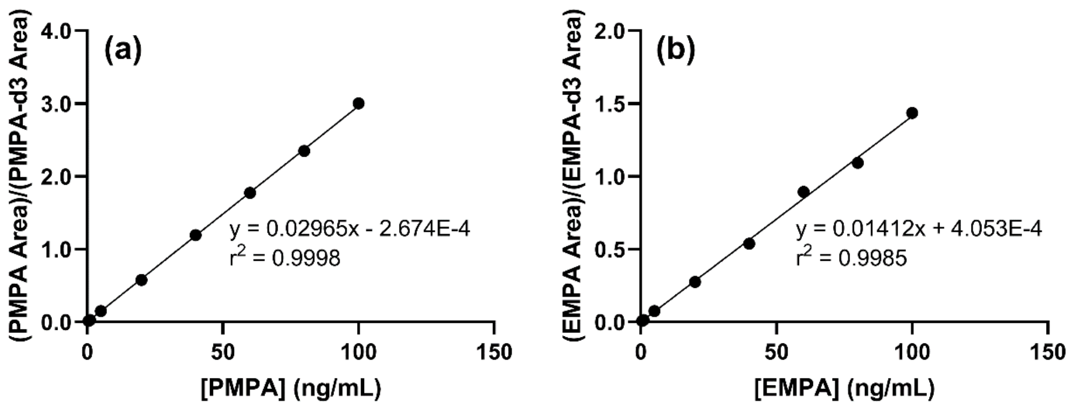

Fig. S1 Representative standard calibration curve with 1/*y* weighting from 0.5 to 100 ng/mL of PMPA (a) and 0.5 to 100 ng/mL of EMPA (b) in human whole blood.

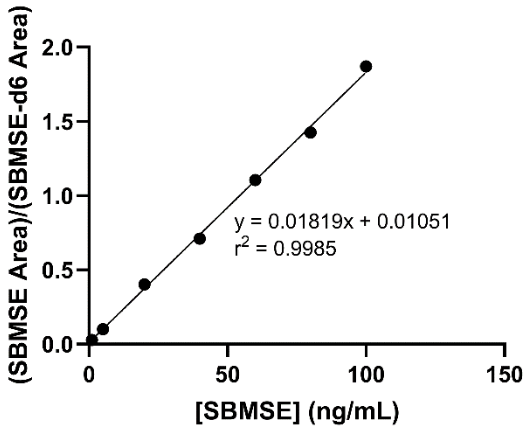

Fig. S2 Representative standard calibration curve with 1/*y* weighting from 1 to 100 ng/mL of SBMSE in human whole blood.

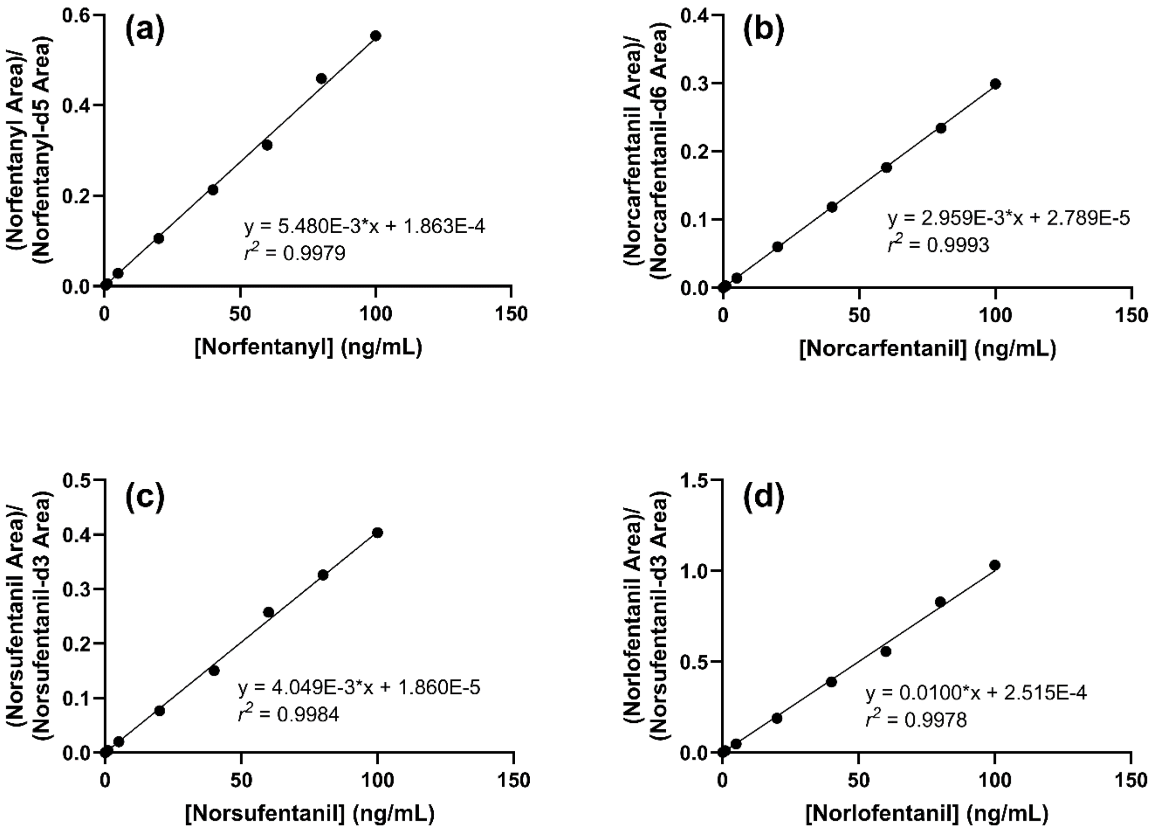

Fig. S3 Representative standard calibration curve with 1/*y* weighting from 0.5 to 100 ng/mL of norfentanyl (a), 0.05 to 100 ng/mL of norcarfentanil (b), 0.05 to 100 ng/mL of norsufentanil (c), and 0.1 to 100 ng/mL of norlofentanil (d) in human whole blood.
